# Sex-dependent regulation of mucin gene transcription and airway secretion and mechanics following intra-airway IL-13 in mice with conditional loss of club cell Creb1

**DOI:** 10.3389/fphys.2024.1392443

**Published:** 2024-04-22

**Authors:** Mariana Sponchiado, Amy Fagan, Luz Mata, Angelina L. Bonilla, Pedro Trevizan-Baú, Sreekala Prabhakaran, Leah R. Reznikov

**Affiliations:** ^1^ Department of Physiological Sciences, University of Florida, Gainesville, FL, United States; ^2^ Department of Pediatrics Pediatric Pulmonary Division, University of Florida, Gainesville, FL, United States

**Keywords:** club cell, IL-13, sex differences, mucin, airway mechanics

## Abstract

**Introduction:** Interleukin 13 (IL-13) is an important effector molecule in allergic asthma. IL-13-mediated mucin hypersecretion requires conversion of secretoglobin-positive club cells into goblet cells through suppression of *forkhead box A2* (*FOXA2*) and induction of *SAM pointed domain containing ETS transcription factor* (*SPDEF*). IL-13-mediated mucin hypersecretion may also include modulation of purinergic and muscarinic receptors that control basal and stimulated mucin secretion. We recently found that the transcription factor cAMP response element-binding protein (Creb1) inhibits *FOXA2* and modulates mucus secretion in mice.

**Methods:** We tested the hypothesis that loss of club cell Creb1 mitigates the pro-mucin effects of IL-13. We challenged male and female mice with conditional loss of club cell Creb1 and wild type littermates with intra-airway IL-13 or vehicle. We also studied human “club cell-like” NCI-H322 cells.

**Results:** Loss of club cell Creb1 augmented IL-13-mediated increases in mRNA for the gel-forming mucins *Muc5ac* and *Muc5b* and prevented IL-13-mediated decreases in *muscarinic 3 receptor* (*M3R*) mRNA in male airways. In female airways, loss of club cell Creb1 reduced *M3R* mRNA and significantly blunted IL-13-mediated increases in *purinergic receptor P2Y2* (*P2ry2*) mRNA but did not impact *Muc5ac* and *Muc5b* mRNA. Despite changes in mucins and secretion machinery, goblet cell density following cholinergic stimulation was not impacted by loss of club cell Creb1 in either sex. IL-13 treatment decreased basal airway resistance across sexes in mice with loss of club cell Creb1, whereas loss of club cell Creb1 augmented IL-13-mediated increases in airway elastance in response to methacholine. NCI-H322 cells displayed IL-13 signaling components, including IL-13Rα1 and *IL-4Rα*. Pharmacologic inhibition of CREB reduced IL-13Rα1 mRNA, whereas recombinant CREB decreased *IL-4Rα* mRNA. Application of IL-13 to NCI-H322 cells increased concentrations of cAMP in a delayed manner, thus linking IL-13 signaling to CREB signaling.

**Conclusion:** These data highlight sex-specific regulation of club cell Creb1 on IL-13-mediated mucin hypersecretion and airway mechanics.

## Introduction

Asthma is one of the most common chronic airway diseases among children (Gershon et al., 2010). Features of asthma include wheezing, chest tightness, cough, mucus hypersecretion and variable airflow obstruction (GINA, 2017). Recent estimates suggest that asthma costs $81.9 billion per year (Nurmagambetov et al., 2018), and in 2015 claimed 338,000 lives (WHO, 2018). There are known sex and gender differences in asthma prevalence, with boys having higher prevalence in childhood and women having higher prevalence in adulthood (Chowdhury et al., 2021).

Enhanced production of mucin 5AC (MUC5AC) due to goblet cell metaplasia, paired with airway remodeling and inflammation, drive airway morbidity and mortality in asthma. For example, the Severe Asthma Research Program found that 58% of people with asthma exhibited airway mucus plugs (Dunican et al., 2018), and the extent of mucus plugging correlated with airflow limitation and worse control of asthma. Additionally, a small imaging study showed that individuals experiencing an asthma exacerbation had >40% of the 4^th^ and 5^th^ generation bronchi obstructed with mucus (Yoshida et al., 2020). Similar findings showing mucus obstruction of the airway due to mucus plugs and airway narrowing have been repeatedly described (Mummy et al., 2022; Tang et al., 2022). Importantly, mucus obstruction can precipitate disease symptoms (i.e., shortness of breath), as well prevent effective ventilation.

Goblet cells and submucosal glands are the major sources of mucins in the airways. However, club cells, which are the primary secretory cell in the small airway and represent ∼15–44% of all proliferating cells in human terminal bronchioles (Boers et al., 1999), also secrete low levels of mucins (Hiemstra et al., 2015). Perhaps more importantly, club cells can differentiate into goblet cells (Chen et al., 2009). The conversion of club cells to goblet cells requires induction of SAM pointed domain containing ETS transcription factor (SPDEF) (Kim et al., 2019), and suppression of forkhead box A2 (FOXA2) (Chang et al., 2000; Chen et al., 2009). The relationship between SPDEF and FOXA2 is inverse (Chen et al., 2009; Chen et al., 2010; Yu et al., 2010; Choi et al., 2020), with SPDEF suppressing FOXA2 (Chen et al., 2009), and FOXA2 suppressing SPDEF (Chen et al., 2010).

The type 2 cytokine interleukin 13 (IL-13) is a critical mediator of allergic asthma (Wills-Karp et al., 1998) and drives goblet cell metaplasia. IL-13 signaling requires binding of IL-13 to the α chain of the IL-4 receptor IL-4Rα and the IL-13 binding receptor α1 (IL-13Rα1), leading to activation of several downstream signaling molecules, including JAK-STAT pathways (Junttila, 2018). This pathway leads to induction of SPDEF (Park et al., 2007) and decreased expression of FOXA2 (Chen et al., 2009). IL-13 also binds IL-13Rα2, which behaves as a decoy receptor that negatively regulates levels of IL-13 (Lupardus et al., 2010).

IL-13 also drives airway hyperreactivity (AHR). The mechanisms responsible are unclear. For example, Kuperman and colleagues demonstrated that when IL-13 signaling is active only in the airway epithelia, it is sufficient to induce AHR (Kuperman et al., 2002). However, others have shown that IL-13 enhances contractility of airway smooth muscles via direct action on airway smooth muscle (Risse et al., 2011). However, IL-13 signaling on airway smooth muscle is also sufficient but not necessary to induce AHR (Perkins et al., 2011). Thus, IL-13-mediated alterations in the airway epithelium, such as mucus production and barrier permeability, may mediate AHR directly, or indirectly through modulation of airway smooth muscle.

We recently demonstrated that the cAMP response element-binding protein (Creb1), a transcription factor in the basic leucine zipper superfamily (Shaywitz and Greenberg, 1999), regulates the goblet cell transcriptional network and mucus secretion in response to IL-1B. This modulation was likely due to direct interaction of CREB with FOXA2, such that inhibition of CREB increased FOXA2 expression and *vice versa* (Sponchiado et al., 2023). Given that IL-13 is a key driver of goblet cell metaplasia and enhanced mucin synthesis through pathways involving SPDEF and FOXA2, we hypothesized that loss of club cell Creb1 mitigates the pro-mucin effects of IL-13.

## Materials and methods

### Animals

Mice with conditional loss of club cell Creb1 were created as previously described by our lab (Sponchiado et al., 2023). Briefly, male and female mice heterozygous for floxed *Creb1* and containing tamoxifen inducible Cre-recombinase under the Secretoglobin Family 1A Member 1 (*Scgb1a1*) promoter were bred. The resultant mice (Creb1^fl/fl^Scgb1a1^+^ and Creb1^fl/fl^Scgb1a1^wt^) were studied. All breeding was performed by the University of Florida Rodent Models breeding core. Adult (8–10 weeks old) male and female mice were studied. All mice were kept on 12 h light/dark cycle, fed *ad libitum* standard chow diet (2918, Teklad) and provided *ad libitum* access to water. Procedures were approved by and adhered to the University of Florida Institutional Animal Care and Use Committee.

### IL-13 and tamoxifen treatment

We used established protocols from our lab (Sponchiado et al., 2023). Briefly, Creb^fl/fl^Scgb1a1^+^ and Creb^l/fl^Scgb1a1^wt^ mice were lightly anesthetized under gaseous isoflurane (2%) in an induction chamber and intranasally administered 50 µL of sterile IL-13 (50 μg/mL) in 0.9% saline or sterile 0.9% saline vehicle using a pipette for four consecutive days (Pezzulo et al., 2019). Delivery of IL-13 in this manner leads to robust goblet cell hypertrophy in mouse lungs (Pezzulo et al., 2019). All mice (Creb1^fl/fl^Scgb1a1^+^ and Creb1^fl/fl^Scgb1a1^wt^) received an intraperitoneal injection containing 100 µL of sterile tamoxifen dissolved in corn oil (20 mg/mL) on days 1 and 3 out of the 4 days IL-13 treatment regimen to induce *Cre*-recombinase activity and excision of floxed *Creb1* as previously described by our lab (Sponchiado et al., 2023).

### FlexiVent

Pulmonary mechanics were evaluated 20–24 h after the last IL-13 administration. Procedures were performed as previously described (Reznikov et al., 2018; Sponchiado et al., 2023). Briefly, a tracheotomy was performed in anesthetized mice (ketamine/xylazine/acepromazine) and a cannula (blunted 18 g needle) was inserted into the trachea. Mice were ventilated at 150 breaths/min at a volume of 10 mL/kg of body mass and administered a paralytic (rocuronium bromide). Increasing doses of methacholine from 12.5 to 100 mg/mL were aerosolized using an ultrasonic nebulizer for a 10 s duration as we previously described (Reznikov et al., 2018). Anesthetized animals were euthanized at the end of the flexiVent protocol via cervical dislocation.

### Bronchoalveolar lavage and analyses

Three sequential 1 mL lavages of 0.9% sterile saline were delivered into the airway *postmortem*. All collected material from 1 mouse was pooled, spun at 500 x g, and the supernatant removed and frozen at −80°C. Cells were counted on a hemocytometer.

### Enzyme-linked immunosorbent assay

ELISAs for murine Muc5ac (M7906) and murine Muc5b (M7978) were purchased from Biotang (Lexington, MA, United States). BAL samples were run in duplicate and read using a filter-based accuSkan FC microplate photometer (ThermoFisher Scientific, Waltham, MA, United States). Concentrations were determined by an 8-point standard curve ranging from 0.625 to 80 ng/mL plotted in a 4-parameter logistic sigmoidal curve (*R* ^2^ > 0.99). Muc5ac and Muc5b protein standards were provided in the kits. The limits of sensitivity were 0.3 ng/mL for both. The intra-assay coefficients of variability were 8.89% and 6.88% for Muc5ac and Muc5b, respectively.

### Histology

Following flexiVent procedures and exposure to the methacholine dose response curve, the left lung was removed and placed in 10% normal buffered formalin. Lungs were not pressure inflated or actively perfused with fixative. Standardized procedures were established in which lungs were embedded in paraffin and transversely sectioned starting at airways most distal to the bronchus. All lungs were embedded and sectioned in standardized fashion with the ventral lobar surfaces oriented down in the cassette. Paraffin-embedded samples were sectioned at 4 μm thickness transversely through the terminal bronchioles and lower bronchioles. Sections containing lower bronchioles were selected and stained with Alcian Blue/Periodic Schiff (PAS stain) (Epredia, cat. #87023), according to the manufacturer’s instructions. Airways were imaged using a Zeiss Axio Zoom.V16 (Carl Zeiss, Germany) microscope. Alcian Blue/PAS-positive cells were counted independently by two masked observers and normalized to airway luminal area using ZenPro software (Carl Zeiss). Alcian Blue/PAS-positive cell numbers were averaged across observers and the mean per mouse was used for statistical analysis.

### Immunohistochemistry

Paraffin-embedded lung sections (4 μm) were deparaffinized and endogenous peroxidase activity blocked with 3% hydrogen peroxide in methanol for 30 min. Sections were incubated in 10 mM sodium citrate buffer (pH 6) and microwaved for antigen retrieval. MUC5B (rabbit anti-MUC5B; HPA008246, Millipore Sigma; 1:500 dilution; 2 h) immunolabeling was performed using the Vectastain Elite ABC system (PK-6101, Vector Laboratories, CA, United States) according to the manufacturer’s protocol. Staining was developed with 3,3′-diaminobenzidine tetrachloride (34,065, ThermoFisher Scientific). Samples were counterstained with hematoxylin. Images were captured with a Zeiss Axio Zoom.V16 (Carl Zeiss) microscope. Muc5b mean intensity in central airways was semi-quantified by tracing the epithelial area and using the IHC plugin in ImageJ (http://rsb.info.nih.gov/ij/).

### NCI-H322 cell culture and treatment

Human NCI-H322 cells were obtained from the European Collection of Authenticated Cell Cultures (ECACC; Sigma). Ultrastructural studies of this male bronchoalveolar adenocarcinoma-derived cell line demonstrated the presence of cytoplasmic structures characteristic of club cells (Lau et al., 1987; Schuller et al., 1991). NCI-H322 cells were grown in RPMI medium (11,875–093; Gibco) supplemented with 10% fetal bovine serum (26,140–079; Gibco) and 1% penicillin/streptomycin (15,140–122; Gibco). Cultures were maintained in humid atmosphere at 37°C and 5% CO_2_.

#### CREB inhibitor experiment

Cells (passage 105) were seeded onto 12-well plates. At subconfluency (90%), monolayers were assigned to the following treatments: 1) 100 nM 666–15 [CREB inhibitor (Li et al., 2016)] (*n* = 6) or 2) vehicle control (*n* = 6). The medium was renewed every 24 h with above treatments for four consecutive days. After 4 days of treatment, cells were harvested with QIAzol (Qiagen, Hilden, Germany), snap frozen and stored at −80°C until RNA isolation was performed. The dose of 666–15 used in this study is below the dose that elicits off-target effects [off-target effects observed at > 2 μM *in vivo* (Li et al., 2016)].

#### NCI-H322 cells treated with recombinant human CREB protein

Methods are identical to those described by our lab (Sponchiado et al., 2023). Briefly, NCI-H322 cells were seeded at 250,000 cells per well in a 24-well plate. Using data provided by Novus Biologics website, we estimated that 10,000,000 cells contain approximately 10 ng of CREB. Therefore, to double the approximated amount of CREB available to a cell, we treated −250,000 cells with 250 pg of human recombinant CREB (in addition to the −250 pg of endogenous CREB). To do this, CREB (H00001385; Novus Biologicals) was resuspended in complete media (*n* = 8). Vehicle control was 50 mM Tris-HCl (pH 8.0) diluted in complete media to a final concentration of 1.25 mM Tris-HCl (to match the final concentration of Tris-HCl in the recombinant CREB treatments group) (*n* = 8). The medium was renewed every 24 h with above treatments for four consecutive days. After 4 days of treatment, cells were harvested with QIAzol (Qiagen, Hilden, Germany), snap frozen and stored at −80°C until RNA isolation.

#### IL-13 treatment experiments to assess cAMP levels

NCI-H322 cells were seeded at 10,000 cells per well as described in the manufacturers protocol in a 96-well plate with 1) 6.25 ng/mL IL-13 (*n* = 8); 2) 12.5 ng/mL IL-13 (*n* = 8); 3) 25 ng/mL IL-13 (*n* = 8) or 4) vehicle control (*n* = 6) for 30 min. Doses were chosen based upon prior data demonstrating induction of SPDEF and MUC5AC in human bronchial epithelial cells (Yu et al., 2010) and robust goblet cell metaplasia in human primary airway epithelia (Pezzulo et al., 2019). The 30-min time point was based upon prior work in human monocytes showing acute increases in cAMP upon application of IL-13 (Sozzani et al., 1995). A second experiment with identical treatments was conducted with incubation lasting for 8 h. An 8- h time point was conducted to capture potential indirect mechanisms leading to cAMP accumulation, as observed in prior work (Sponchiado et al., 2023). Details about the cAMP assay are in below section.

### Measurement of cAMP

The cAMP-Glo Max Assay (Promega, cat. #V1681) was followed according to the manufacturer’s instructions and as previously described by our lab (Sponchiado et al., 2023). Briefly, NCI-H322 cells were seeded at a density of 10,000 cells/well in 96-well clear bottom assay plate per the manufacturer’s protocol (Corning, cat. #3610). The next day, cells were stimulated with IL-13 or vehicle control (as detailed above). A standard curve was constructed according to the manufacturer’s instructions. The plate was read on an Agilent Bio Tek Syngery LX multi-mode reader using endpoint luminescence protocol. The cAMP concentrations for vehicle and IL-13-treated NCI-H322 cells were calculated using the values from the standard curve and plotted in a sigmoidal 4PL curve (GraphPad Prism 9) as we previously described (Sponchiado et al., 2023).

### RNA isolation and qRT-PCR

RNA from the cranial right lung lobe and human NCI-H322 cells was isolated using RNeasy Lipid Tissue kit (Qiagen) with in-column DNase digestion (Qiagen). RNA concentration was measured with a NanoDrop (ThermoFisher Scientific). Total RNA (2000 ng) was reverse transcribed using Superscript VILO Master Mix (ThermoFisher Scientific). Transcripts for *Muc5ac*, *Muc5b*, purinergic receptor P2Y2 (*P2ry2),* member RAS oncogene family *(Rab3D),* and cholinergic receptor muscarinic 3 *(M3R)* were quantified in mouse lung homogenates using primers designed in PrimerQuest (IDT; idtdna.com) and based on *Mus musculus* mRNA GenBank (NCBI; www.ncbi.nlm.nih.gov) sequences. Transcripts for *IL-13Rα1*, *IL-4Rα*, and *IL-13Rα2* were quantified in NCI-H322 cells using primers based on *Homo sapiens* mRNA GenBank sequences. All primers are listed is Supplementary Table S1. PCR reactions were carried out in triplicates in 96-well plates with fast SYBR Green master mix (Applied Biosystems, Waltham, MA, United States). PCR parameters included denaturation at 95°C for 10 min, followed by 45 cycles of 10 s at 95°C, annealing at 60°C for 10 s, and extension at 72°C for 10 s. A melting curve was performed at the end to confirm presence of single amplicons. Primer pair specificity was further confirmed by electrophoresis of single band PCR products. Relative abundances were calculated using the 2^−ΔΔCT^ method (Livak and Schmittgen, 2001). Actin beta (*Actb*) was used as endogenous control for mouse lung samples, and ribosomal protein L13a (*RPL13A*) for human cells.

### Chemicals and drugs

Acetyl-beta-methacholine-chloride (Sigma) was dissolved in 0.9% saline for flexiVent studies. Mouse IL-13 (R&D Systems, MN, United States) was dissolved in 0.9% sterile saline containing 0.1% BSA carrier to a concentration of 50 μg/mL, aliquoted and stored at −20°C until use. Fresh aliquots were thawed on the day of treatment for intranasal delivery each day. The CREB inhibitor 666–15 (Li et al., 2016) (R&D Systems) was initially dissolved in 100% DMSO at a concentration of 1 mM. The stock was diluted 1:10,000 into complete growth media for cell culture experiments.

### Statistical analysis

For basal flexiVent measurements, a three-way ANOVA was performed with sex, genotype and treatment as the main factors and the resulting double and triple interactions. A three-way ANOVA was performed for flexiVent studies with methacholine dose as a repeated measure, and genotype and treatment as main factors and the resulting double and triple interactions. For many studies, sexes were analyzed separately given known sex differences in allergic asthma (Chowdhury et al., 2021) and a two-way ANOVA was performed with genotype and treatment as main factors. Significance for main effects and interactions was set at *p* < 0.05. Post hoc comparisons were performed using a Sidak’s multiple comparisons test; major comparisons of interest were: 1) Creb1^fl/fl^Scgb1a1^wt^ + IL-13 vs. Creb1^fl/fl^Scgb1a1^wt^ + vehicle; and 2) Creb1^fl/fl^Scgb1a1^cre^ + IL-13 vs. Creb1^fl/fl^Scgb1a1^cre^ + vehicle. For human cell studies, several independent experiments were performed with two treatment groups; therefore, a student’s unpaired *t*-test was performed with significance set at *p* < 0.05. Statistical analyses were performed using GraphPad Prism 9.0a. Data are presented as mean ± SEM.

## Results

Loss of club cell Creb1 modifies basal airway resistance and augments airway elastance in response to IL-13. In our prior work, we showed club cell recombination rates of −75% (Sponchiado et al., 2023). Here, we confirmed *Cre*-recombinase mRNA expression in Creb1^fl/fl^Scgb1a1^cre^ mice lung homogenates through qRT-PCR. We found average Ct values for *Cre* mRNA of 20.38 ± 0.49 in male lung homogenates (*n* = 6) and average Ct value of 21.35 ± 0.15 in female lung homogenates (*n* = 9), while absent in Creb1^fl/fl^Scgb1a1^wt^ mice. These values were consistent with our previous report (Sponchiado et al., 2023).

**FIGURE 1:**
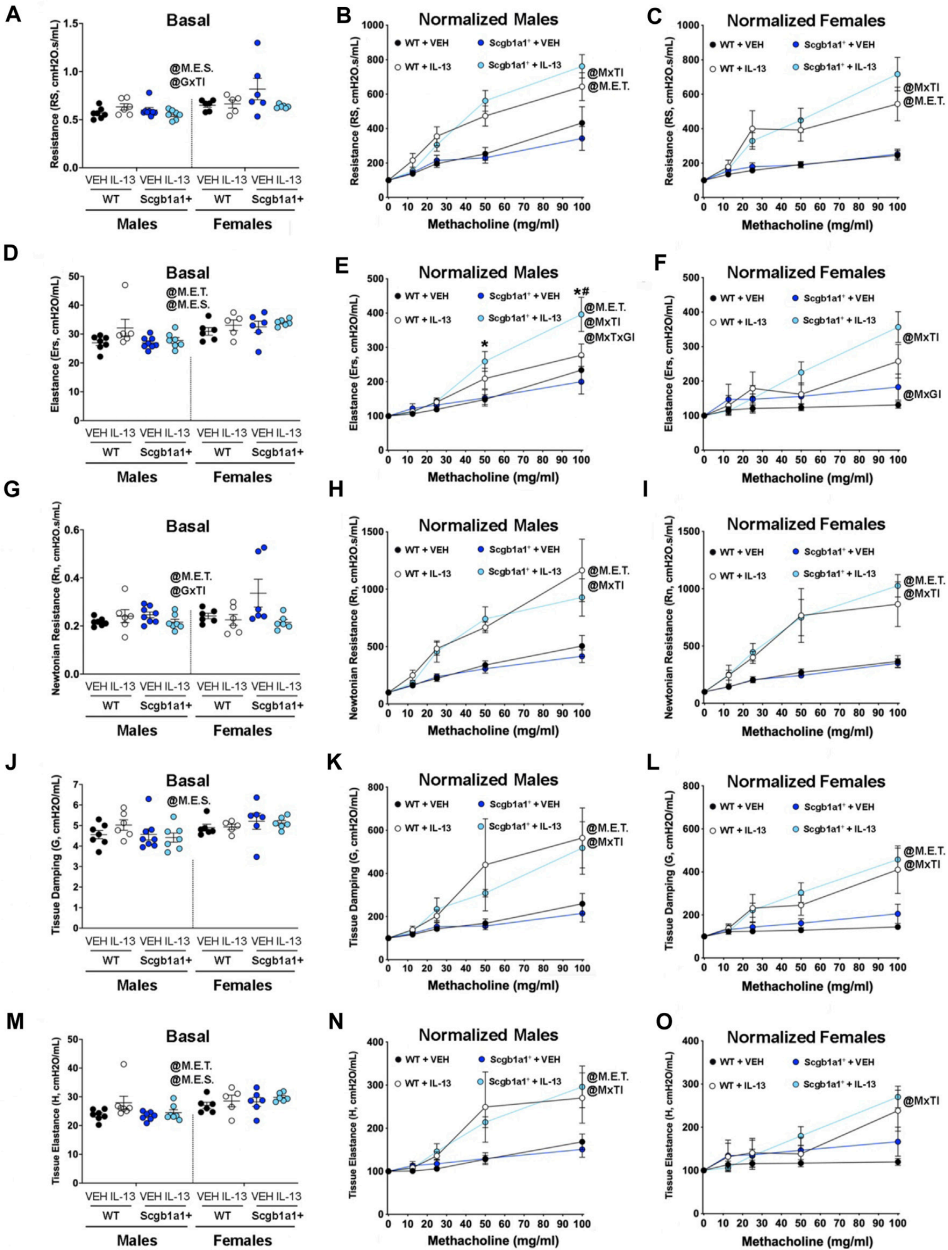
Conditional loss of club cell Creb1 affects IL-13-mediated impairments in resistance and elastance. **(A)** Basal airway resistance. **(B)** Normalized resistance in response to methacholine in male airways. **(C)** Normalized airway resistance in response to methacholine in female airways. **(D)** Basal airway elastance. **(E)** Normalized elastance in male airways in response to methacholine. * = compared to Scgb1a1^+^ + VEH. # compared to WT + IL-13. **(F)**. Normalized elastance in female airways in response to methacholine. **(G)** Basal airway Newtonian resistance. **(H)** Normalized Newtonian resistance in male airways in response to methacholine. **(I)** Normalized Newtonian resistance in female airways in response to methacholine. **(J)** Basal tissue damping. **(K)** Normalized tissue damping in male airways in response to methacholine. **(L)** Normalized tissue damping in female airways in response to methacholine. **(M)** Basal tissue elastance. **(N)** Normalized tissue elastance in male airways in response to methacholine. **(O)** Normalized tissue elastance in female airways in response to methacholine. Males: Creb1^fl/fl^Scgb1a1^wt^ mice treated with vehicle (*n* = 7) or IL-13 (*n* = 6); Creb1^fl/fl^Scgb1a1^+^ mice treated with vehicle (*n* = 8) or IL-13 (*n* = 7). Females: Creb1^fl/fl^Scgb1a1^wt^ mice treated with vehicle (*n* = 6) or IL-13 (*n* = 6); Creb1^fl/fl^Scgb1a1^+^ mice treated with vehicle (*n* = 6) or IL-13 (*n* = 6). Abbreviations: WT, wild type; Scgb1a1^+^, club cell promoter driving CRE recombinase; IL-13, Interleukin 13; VEH, vehicle; @M.E.S., main effect of sex; @GxTI, genotype × treatment interaction; @M.E.T., main effect of treatment; @MxTI, methacholine × treatment interaction; @MxGxTI, methacholine x genotype × treatment interaction; @MxGI, methacholine × genotype interaction. Additional details about *p* values are shown in Table 1.

We first examined the impact of IL-13 administration on airway resistance using single frequency forced oscillation. We found a main effect of sex (*p* = 0.0019, Figure 1A;, Table 1) on basal airway resistance. We also found a statistically significant genotype × treatment interaction in basal airway resistance (*p* = 0.019, Figure 1A; Table 1). This interaction meant that when collapsing data across sexes, loss of club cell Creb1 influenced the impact of IL-13 on basal airway resistance (Supplementary Figure S1A). Post hoc analysis corrected by Sidak’s multiple comparison test identified a trend for IL-13 to reduce basal airway resistance in mice with loss of conditional club cell Creb1 (*p* = 0.079, Supplementary Figure S1A), but not in mice with intact Creb1 (*p* = 0.675, Supplementary Figure S1A). When examining the impacts of IL-13 on normalized airway resistance in response to methacholine, we observed a statistically significant methacholine × treatment interaction in both male (*p* < 0.0001, Figure 1B; Table 1) and female (*p* < 0.0001, Figure 1C; Table 1) airways. These results demonstrated that IL-13 induced AHR independent of genotype, and that loss of club cell Creb1 selectively impacted basal airway resistance in response to IL-13.

**TABLE 1:** Summary of statistically significant main effects and interactions for airway mechanic studies.

Sex	Experimental Outcome	Statistical parameter	*p*-value
Both	Basal Airway Resistance	Main Effect of Sex	0.0019
		Genotype × Treatment Interaction	0.019
Males	Normalized Airway Resistance	Main Effect of Treatment	<0.0001
		Methacholine × Treatment Interaction	<0.0001
		Methacholine x Genotype × Treatment Interaction	0.077
Females	Normalized Airway Resistance	Main Effect of Treatment	<0.0001
		Methacholine × Treatment Interaction	<0.0001
Both	Basal Airway Elastance	Main Effect of Sex	0.0004
		Main Effect of Treatment	0.031
Males	Normalized Airway Elastance	Main Effect of Treatment	0.0053
		Methacholine × Treatment Interaction	<0.0001
		Methacholine x Genotype × Treatment Interaction	0.011
Females	Normalized Airway Elastance	Methacholine × Treatment Interaction	<0.0001
		Methacholine × Genotype Interaction	0.024
Both	Basal Newtonian Resistance	Main Effect of Treatment	0.039
		Genotype × Treatment Interaction	0.022
Males	Normalized Newtonian Resistance	Main Effect of Treatment	<0.0001
		Methacholine × Treatment Interaction	<0.0001
Females	Normalized Newtonian Resistance	Main Effect of Treatment	<0.0001
		Methacholine × Treatment Interaction	<0.0001
Both	Basal Tissue Damping	Main Effect of Sex	0.032
Males	Normalized Tissue Damping	Main Effect of Treatment	0.003
		Methacholine × Treatment Interaction	<0.0001
Females	Normalized Tissue Damping	Main Effect of Treatment	0.001
		Methacholine × Treatment Interaction	<0.0001
Both	Basal Tissue Elastance	Main Effect of Sex	0.0006
		Main Effect of Treatment	0.031
Males	Normalized Tissue Elastance	Main Effect of Treatment	0.002
		Methacholine × Treatment Interaction	<0.0001
Females	Normalized Tissue Elastance	Methacholine × Treatment Interaction	<0.0001

Single frequency forced oscillation also provides information about airway elastance (the inverse of compliance). We found a main effect of IL-13 treatment on basal elastance values (*p* = 0.031, Figure 1D; Table 1), indicating that IL-13 increased basal elastance values across genotype and sex when groups were collapsed (collapsed data not shown). We also found a main effect of sex (*p* = 0.0004, Figure 1D; Table 1). A methacholine x genotype × treatment interaction was observed in males for normalized airway elastance (*p* < 0.0001, Figure 1E; Table 1). The three-way interaction allowed us to perform *post hoc* comparisons where we found that loss of club cell Creb1 augmented the effect of IL-13 at higher methacholine doses in male airways (Figure 1E). A similar effect for loss of club cell Creb1 was observed in female airways, such that a significant methacholine × treatment interaction (*p* < 0.0001) and a significant methacholine × genotype interaction (*p* = 0.024) were observed (Figure 1F; Table 1). The lack of a three-way interaction for normalized airway elastance in females negated the ability to look at differences across genotype and treatment and methacholine doses, like we did for males. These results suggested that loss of club cell Creb1 augmented the effect of IL-13 on airway elastance in response to methacholine independent of sex but did not impact the effects of IL-13 on basal airway elastance values.

We next examined three additional airway mechanic properties in response to broadband frequency forced oscillation: Newtonian resistance, tissue damping, and tissue elastance. Newtonian resistance is the resistance of the central and conducting airways. We found a significant genotype × treatment interaction on basal Newtonian airway resistance (*p* = 0.022, Figure 1G; Table 1). The interaction indicated that genotype influenced the effect of IL-13 on basal Newtonian resistance when data were collapsed across sexes (Supplementary Figure S1B). Post hoc analysis corrected by Sidak’s multiple comparison test indicated that IL-13 reduced basal airway resistance in mice with loss of conditional club cell Creb1 (*p* = 0.013, Supplementary Figure S1B), whereas IL-13 has no effect on basal Newtonian resistance in mice with intact Creb1 (*p* = 0.975, Supplementary Figure S1B). These data indicated that loss of club cell Creb1 selectively impacted basal airway Newtonian resistance in response to treatment. A methacholine × treatment interaction was also found for normalized Newtonian resistance in both male airways (*p* < 0.0001, Figure 1H; Table 1) and female airways (*p* < 0.0001, Figure 1I; Table 1), with IL-13 increasing airway resistance of the central and conducting airways independent of genotype.

Tissue damping is the resistance of the tissue and related to energy dissipation in the alveoli. We found a main effect of sex on basal tissue damping, with males having lower tissue damping across genotype and treatment compared to females (*p* = 0.032, Figure 1J; Table 1). Both male (*p* < 0.0001, Figure 1K; Table 1) and females (*p* < 0.0001, Figure 1L; Table 1) demonstrated methacholine × treatment interactions in normalized tissue damping. These data demonstrated that neither IL-13 nor loss of club cell Creb1 impacted basal tissue damping. Further they indicated that loss of club cell Creb1 has no effect on IL-13-mediated increases in normalized tissue damping in response to methacholine in either sex.

The last parameter examined was tissue elastance, a measure of energy conservation in the alveoli. We found a main effect of sex (*p* = 0.0006) and a main effect of treatment (*p* = 0.031) on basal tissue elastance (Figure 1M; Table 1). The main effect of sex revealed that when collapsing data across genotype and treatment, that female murine airways displayed elevated basal tissue damping levels compared to males (collapsed data not shown). The main effect of treatment indicated that IL-13 increased basal tissue elastance when data were collapsed across sex and genotype (collapsed data not shown). Both male (*p* < 0.0001, Figure 1N; Table 1) and female (*p* < 0.0001, Figure 1O; Table 1) mice showed a significant methacholine × treatment interaction in normalized tissue elastance, with IL-13 increasing tissue elastance in both sexes. The lack of a three-way interaction among methacholine, treatment and genotype prevented us from performing *post hoc* comparisons across treatment groups and genotypes at methacholine doses. Thus, these data demonstrated that loss impact IL-13-mediated increases in tissue elastance in response to methacholine.

### Club cell Creb1 regulates transcription of mucin and secretion machinery in response to IL-13

**FIGURE 2:**
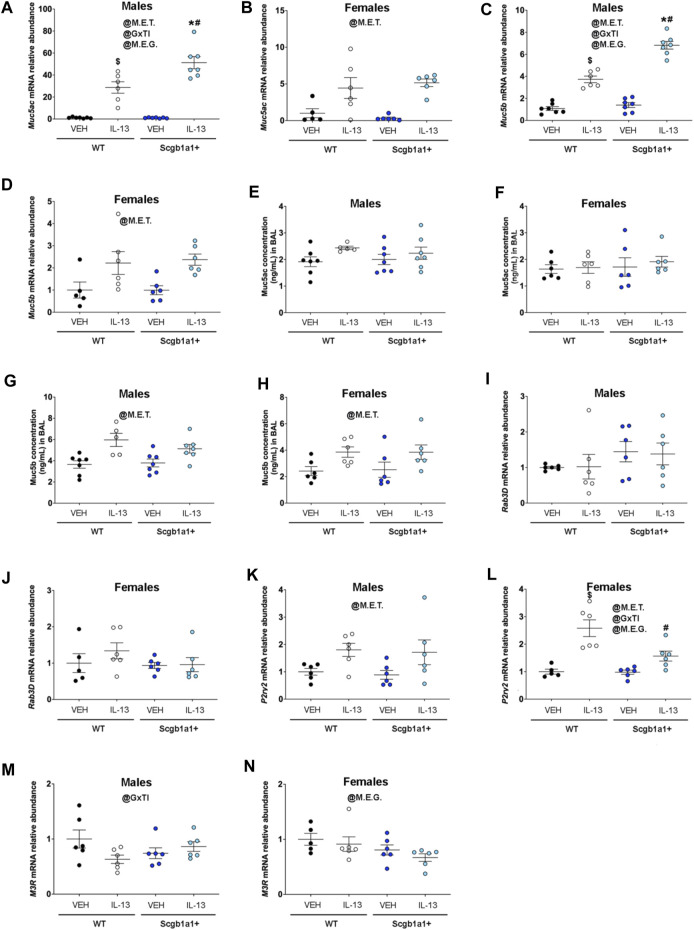
Club cell Creb1 regulates IL-13-mediated mucin synthesis and secretion dynamics. **(A)** *Muc5ac* mRNA in male airways. * = compared to Scgb1a1^+^ + VEH. # compared to WT + IL-13; $ compared to WT + VEH. **(B)** *Muc5ac* mRNA in female airways. **(C)** *Muc5b* mRNA in male airways. * = compared to Scgb1a1^+^ + VEH. # compared to WT + IL-13; $ compared to WT + VEH. **(D)** *Muc5b* mRNA in female airways. **(E)** Muc5ac protein concentration in the BAL of male airways. **(F)**. Muc5ac protein concentration in the BAL of female airways. No significant effects of genotype or treatment. **(G)** Muc5b protein concentration in the BAL of male airways. **(H)** Muc5b protein concentration in the BAL of female airways. **(I)** *Rab3D* mRNA in male airways. Trend for main effect of genotype, *p* = 0.092. **(J)** *Rab3D* mRNA in female airways. No significant effects of genotype or treatment. **(K)** *P2y2* mRNA in male airways. **(L)** *P2y2* mRNA in female airways. # compared to WT + IL-13; $ compared to WT + VEH. **(M)** *M3R* mRNA in male airways. **(N)** *M3R* mRNA in female airways. For all panels, individual points are data collected from a single mouse. Male mice: panels A & C, Creb1^fl/fl^Scgb1a1^wt^ mice treated with vehicle (*n* = 7) or IL-13 (*n* = 6); Creb1^fl/fl^Scgb1a1^+^ mice treated with vehicle (*n* = 7) or IL-13 (*n* = 7); panels E & G, Creb1^fl/fl^Scgb1a1^wt^ mice treated with vehicle (*n* = 7) or IL-13 (*n* = 5); Creb1^fl/fl^Scgb1a1^+^ mice treated with vehicle (*n* = 7) or IL-13 (*n* = 7); panels I, K & M, Creb1^fl/fl^Scgb1a1^wt^ mice treated with vehicle (*n* = 6) or IL-13 (n = 6); Creb1^fl/fl^Scgb1a1^+^ mice treated with vehicle (*n* = 6) or IL-13 (*n* = 6). Female mice: panels B & D, Creb1^fl/fl^Scgb1a1^wt^ mice treated with vehicle (*n* = 5) or IL-13 (*n* = 6); Creb1^fl/fl^Scgb1a1^+^ mice treated with vehicle (*n* = 6) or IL-13 (*n* = 6); panels F & H, Creb1^fl/fl^Scgb1a1^wt^ mice treated with vehicle (*n* = 6) or IL-13 (*n* = 6); Creb1^fl/fl^Scgb1a1^+^ mice treated with vehicle (*n* = 6) or IL-13 (*n* = 6); panels J, L & N, Creb1^fl/fl^Scgb1a1^wt^ mice treated with vehicle (*n* = 5) or IL-13 (*n* = 6); Creb1^fl/fl^Scgb1a1^+^ mice treated with vehicle (*n* = 6) or IL-13 (*n* = 6). Abbreviations: WT, wild type; Scgb1a1^+^, club cell promoter driving CRE recombinase; IL-13, Interleukin 13; VEH, vehicle; @M.E.G., main effect of genotype; @M.E.T., main effect of treatment; @GxTI, genotype × treatment interaction. Additional details about *p* values are shown in Table 2.

Our studies examining airway mechanics revealed that loss of club cell Creb1 modulated the effects of IL-13 on three major airway properties: total airway resistance (basal values), total airway elastance (in response to methacholine), and Newtonian airway resistance (basal values). Since airway resistance is a measurement of airway narrowing and is influenced by airway smooth muscle and mucus accumulation, we next investigated the impact of loss of club cell Creb1 on two major secreted gel-forming mucins in the airways, *Muc5ac* and *Muc5b.* In male lung homogenates, we found a significant treatment × genotype interaction (*p* = 0.005) for *Muc5ac* mRNA expression (Figure 2A; Table 2). The interaction allowed for *post hoc* comparisons, in which we observed that IL-13 increased *Muc5ac* mRNA in wild type mice and mice with loss of club cell Creb1, but to a greater magnitude in mice with loss of club cell Creb1*.* A main effect of treatment (*p* < 0.0001) was observed for *Muc5ac* mRNA in female lung homogenates, though loss of club cell Creb1 had no impact (Figure 2B; Table 2). Similar patterns to *Muc5ac* were observed for *Muc5b* mRNA, with a genotype × treatment interaction (*p* < 0.0001) observed in male lung homogenates (Figure 2C; Table 2). Post hoc comparisons revealed augmentation of the effect of IL-13 on *Muc5b* mRNA in male mice with loss of club cell Creb1. Only a main effect of treatment (*p* = 0.002) on *Muc5b* mRNA was observed in female lung homogenates (Figure 2D; Table 2). These data indicated that IL-13 enhanced transcription of both *Muc5ac* and *Muc5b* mRNA in male and female mice. However, loss of club cell Creb1 augmented the effects of IL-13 in male mice only and had no impact on the effects of IL-13 in female mice.

**TABLE 2:** Summary of statistically significant main effects and interactions for airway mucin and secretion machinery studies.

Sex	Experimental Outcome	Statistical parameter	*p*-value
Males	Muc5ac mRNA	Main Effect of Genotype	0.006
		Methacholine x Treatment	<0.0001
		Genotype × Treatment Interaction	0.005
Females	Muc5ac mRNA	Main Effect of Treatment	<0.0001
Males	Muc5b mRNA	Main Effect of Genotype	<0.0001
		Main Effect of Treatment	<0.0001
		Genotype × Treatment Interaction	<0.0001
Females	Muc5b mRNA	Main Effect of Treatment	0.002
Males	Muc5ac protein in BAL	Trend for Main Effect of Treatment	0.065
Females	Muc5ac protein in BAL	No Main Effects or Interactions	N/A
Males	Muc5b protein in BAL	Main Effect of Treatment	0.0003
Females	Muc5b protein in BAL	Main Effect of Treatment	0.009
Males	Rab3D mRNA	Trend for Main Effect of Genotype	0.092
Females	Rab3D mRNA	No Main Effects or Interactions	N/A
Males	P2y2 mRNA	Main Effect of Treatment	0.008
Females	P2y2 mRNA	Main Effect of Treatment	<0.0001
		Main Effect of Genotype	0.015
		Genotype × Treatment Interaction	0.019
Males	M3R mRNA	Genotype × Treatment Interaction	0.041
Females	M3R mRNA	Main Effect of Genotype	0.047

To determine if parallel increases in mucin proteins were observed, we measured concentrations of Muc5ac and Muc5b in the bronchioalveolar lavage fluid. We found a trend for main effect of IL-13 to increase Muc5ac protein in male airways (*p* = 0.065, Figure 2E; Table 2) with no effect of loss of club cell Creb1. In female airways, we saw no effect of either treatment or genotype on Muc5ac protein concentrations (Figure 2F). Both male (*p* = 0.0003, Figure 2G; Table 2) and female (*p* = 0.009, Figure 2H; Table 2) airways showed significant main effects of IL-13 to increase Muc5b protein concentrations in the bronchoalveolar lavage fluid with genotype having no effect.

Since lavage protein concentrations for Muc5ac in response to IL-13 were not increased despite increased *Muc5ac* mRNA levels, we hypothesized that mucin secretion mechanisms may be impacted. Therefore, we measured the mRNA expression of three molecules that regulate mucin secretion. We first measured mRNA expression of *Rab3D*, a molecule important for mucin vesicle docking expressed in club cells (Evans et al., 2004) and goblet cells (Tuvim et al., 2009). We found a trend for loss of club cell Creb1 to increase *Rab3D* mRNA in male airways (*p* = 0.092, Figure 2I; Table 2), but not in female airways (Figure 2J; Table 2).

We also measured the mRNA expression of the *P2Y2 purinergic receptor* (*P2ry2*), which controls mucin secretion under basal conditions in response to ATP (Adler et al., 2013). A main effect of IL-13 to increase *P2ry2* mRNA was observed in male (*p* = 0.008, Figure 2K; Table 2) and female (*p* < 0.0001, Figure 2L; Table 2) airways. However, in female airways, a genotype × treatment interaction was observed (*p* = 0.019), such that loss of club cell Creb1 blunted the effect of IL-13 on *P2ry2* mRNA levels (Figure 2L; Table 2). This finding suggested that basal and ATP-mediated secretion of mucins may be increased in male and female airways following repeated IL-13 administration, but that loss of club cell Creb1 may blunt this effect in female airways.

Lastly, we measured mRNA expression of the *cholinergic muscarinic 3 receptor* (*M3R*), a key regulator of airway mucus secretion and airway smooth muscle contraction in response to cholinergic agonists (Rogers, 2001). We found a significant genotype × treatment interaction in male airways (*p* = 0.041, Figure 2M; Table 2) with IL-13 decreasing *M3R* mRNA in wild type mice but not in mice with loss of club cell Creb1. Despite a significant interaction, *post hoc* analysis corrected by Sidak’s multiple comparison test only revealed a trend for statistical significance between saline- and IL-13-treated wild type animals (*p* = 0.059) and not in mice with conditional loss of club cell Creb1. Interestingly, in female airways, we found a main effect for loss of club cell Creb1 to decrease *M3R* mRNA (*p* = 0.047, Figure 2N; Table 2). These data suggested that repeated IL-13 administration in male airways may impair cholinergic-mediated secretion of mucins, and that loss of club cell Creb1 may protect against this. Conversely, we found no evidence that repeated IL-13 administration impairs cholinergic-mediated mucin secretion in female airways, but rather loss of club cell Creb1 might.

### Loss of club cell Creb1 does not impact the density of goblet cells or the percentage of granulocytes

**FIGURE 3:**
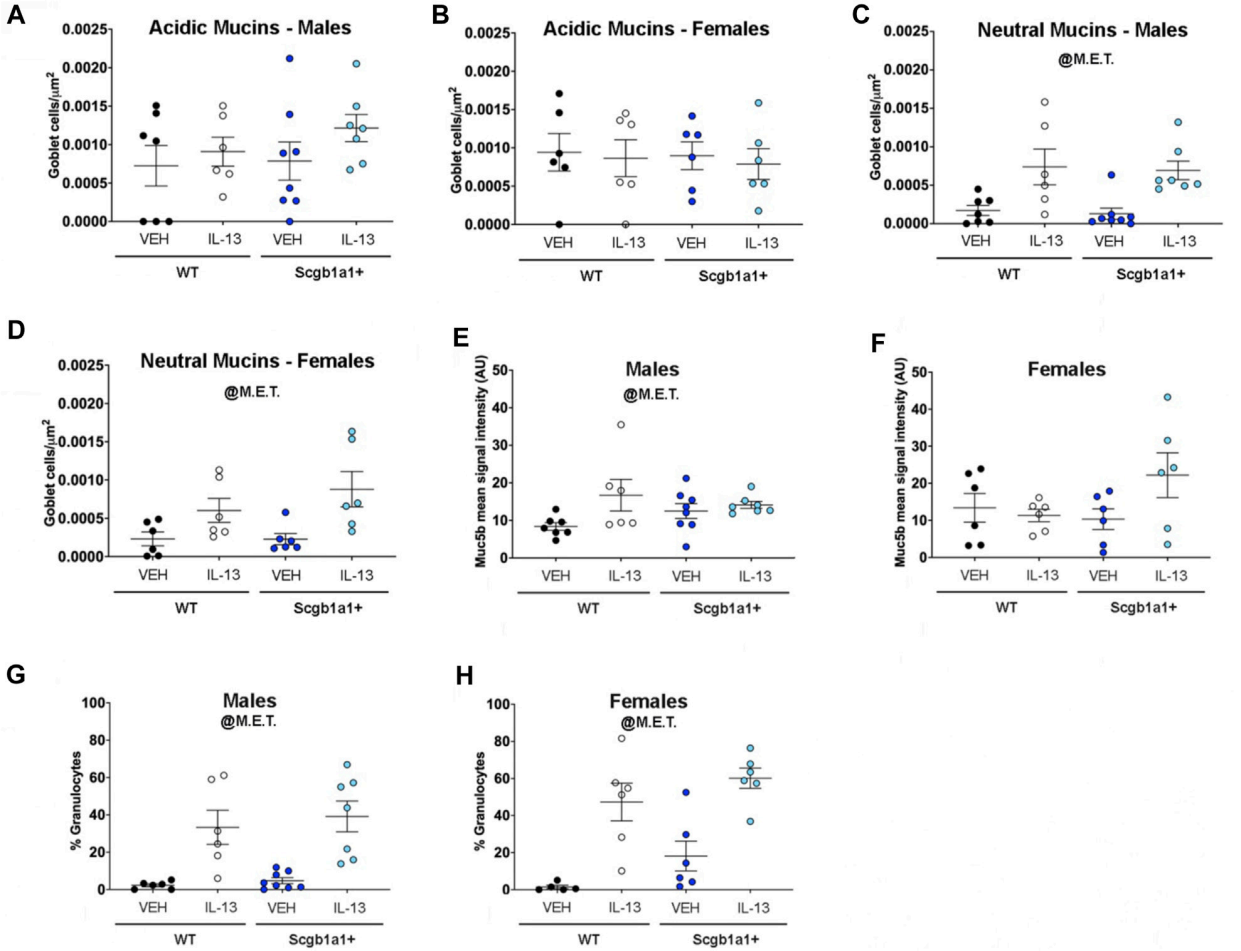
IL-13-mediated goblet cell density is not affected by conditional loss of club cell Creb1. **(A)** Density of goblet cells staining for Alcian-Blue/PAS post methacholine-stimulated conditions in male airways. Density is for cells with acidic mucins, as detected by dark blue staining. No main effects of treatment or genotype were observed. **(B)** Density of goblet cells staining for acidic mucins in female airways. No main effects of treatment or genotype were observed. **(C)** Density of goblet cells staining for neutral mucins, as detected by purple/magenta staining in male airways. **(D)** Density of goblet cells staining for neutral mucins in female airways. **(E)** Mean immunohistochemistry signal intensity of Muc5b in the airway surface post methacholine stimulation in male airways. **(F)** Mean immunohistochemistry signal intensity of Muc5b in the airway surface post methacholine stimulation in female airways. **(G)** Percentage of cells that were granulocytes in the BAL of male mice. **(H)** Percentage of cells that were granulocytes in the BAL of female mice. For all panels, individual points are data collected from a single mouse. Male mice: panels A, C, & E Creb1^fl/fl^Scgb1a1^wt^ mice treated with vehicle (*n* = 7) or IL-13 (*n* = 6); Creb1^fl/fl^Scgb1a1^+^ mice treated with vehicle (*n* = 8) or IL-13 (*n* = 7); panel G, males: Creb1^fl/fl^Scgb1a1^wt^ mice treated with vehicle (*n* = 6) or IL-13 (*n* = 6); Creb1^fl/fl^Scgb1a1^+^ mice treated with vehicle (*n* = 8) or IL-13 (*n* = 7). Female mice: panels B, D & F, Creb1^fl/fl^Scgb1a1^wt^ mice treated with vehicle (*n* = 6) or IL-13 (*n* = 6); Creb1^fl/fl^Scgb1a1^+^ mice treated with vehicle (*n* = 6) or IL-13 (*n* = 6); panel H, Creb1^fl/fl^Scgb1a1^wt^ mice treated with vehicle (*n* = 5) or IL-13 (*n* = 6); Creb1^fl/fl^Scgb1a1^+^ mice treated with vehicle (*n* = 6) or IL-13 (*n* = 6). Abbreviations: WT, wild type; Scgb1a1^+^, club cell promoter driving CRE recombinase; IL-13, Interleukin 13; VEH, vehicle; @M.E.T., main effect of treatment. Additional details about *p* values are shown in Table 3.

Since loss of club cell Creb1 modified mRNA transcription of molecules important for regulated mucin secretion under basal (*P2ry2*) and cholinergic stimulated (*M3R*) conditions, we anticipated that changes in mucus secretion properties might be detected by measuring goblet cell density. Thus, we measured goblet cell density using Alcian-Blue/PAS staining in lung sections from mice post intra-airway methacholine that occurred during airway mechanic (flexiVent) studies. As described in our previous work (Sponchiado et al., 2023), we separated goblet cell density into two groups: cells with acidic mucins, as distinguished with dark blue staining, or cells with neutral mucin, as distinguished with purple staining. The density of goblet cells containing acidic mucins did not differ across genotype or treatment in either sex (Figures 3A,B; Table 3). The density of goblet cells with neutral mucins was increased by IL-13 independent of genotype in both male (*p* = 0.0002, Figure 3C; Table 3) and female (*p* = 0.003, Figure 3D; Table 3) airways.

**TABLE 3:** Summary of statistically significant main effects and interactions for goblet cell, immunohistochemical, and granulocyte analysis.

Sex	Experimental Outcome	Statistical parameter	*p*-value
Males	Goblet Cells—Acidic Mucins	No Main Effects or Interactions	N/A
Females	Goblet Cells—Acidic Mucins	No Main Effects or Interactions	N/A
Males	Goblet Cells—Neutral Mucins	Main Effect of Treatment	0.0002
Females	Goblet Cells—Neutral Mucins	Main Effect of Treatment	0.003
Males	Muc5b immunohistochemistry signal intensity on airway surface	Main Effect of Treatment	0.034
Females	Muc5b immunohistochemistry signal intensity on airway surface	Trend for Genotype × Treatment Interaction	0.092
Males	Percentage of cells that were granulocytes	Main Effect of Treatment	<0.0001
Females	Percentage of cells that were granulocytes	Main Effect of Treatment	<0.0001
		Trend for Main Effect of Genotype	0.059

As described in our previous work (Sponchiado et al., 2023), we performed immunohistochemistry on lung sections. We focused on Muc5b since it is constitutively expressed (Roy et al., 2014). We found a main effect of treatment in male airways, with IL-13 increasing Muc5b protein levels on the airway surface (*p* = 0.0344, Figure 3E; Table 3). A trend for a genotype × treatment interaction was noted in female airways (*p* = 0.092, Figure 3F; Table 3). These results indicated that despite club cell Creb1-mediated regulation of mucin mRNA and mucin secretion machinery, the density of goblet cells in response to IL-13 was not impacted by loss of club cell Creb1*.* However, the results did suggest that in female airways, the retention and/or release of Muc5b may be impacted by loss of club cell Creb1.

We previously reported that loss of club cell Creb1 decreased the number of granulocytes in the mouse airway in response to IL-1B (Sponchiado et al., 2023). To determine whether loss of club cell Creb1 regulated the basic inflammatory response to IL-13, we assessed the percentage of cells that were granulocytes in the bronchoalveolar lavage fluid. We found a main effect of treatment to increase the percentage of cells that were granulocytes independent of genotype in both male (*p* < 0.0001, Figure 3G; Table 3) and female (*p* < 0.0001, Figure 3H; Table 3) airways. However, interestingly, we found a strong trend for loss of club cell Creb1 to augment the percentage of cells that were granulocytes in female airways (*p* = 0.059, Figure 3H; Table 3). These data indicated that loss of club cell Creb1 did not lessen the number of granulocytes infiltrating the airways in response to IL-13 but rather had the potential to augment the percentage of granulocytes in female airways.

### IL-13 increases cAMP in human club cell-like cells

**FIGURE 4:**
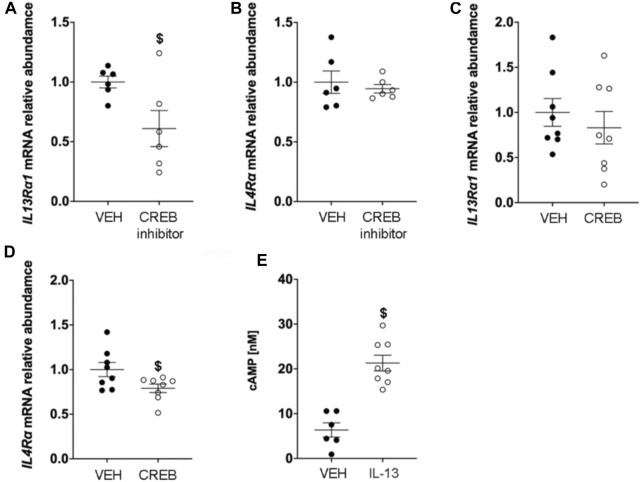
IL-13 increases cAMP concentrations and CREB regulates IL-13 receptor expression. **(A)** Abundance of *IL13Rα1* mRNA in H322 cells treated with vehicle or CREB inhibitor. $ compared to VEH, *p* = 0.034. **(B)** Abundance of *IL4Rα* mRNA in H322 cells treated with vehicle or CREB inhibitor. **(C)** Abundance of *IL13Rα1* mRNA in H322 cells treated with vehicle or recombinant CREB protein. **(D)** *IL4Rα* mRNA in H322 cells treated with vehicle or recombinant CREB protein. $ compared to VEH, *p* = 0.0389. **(E)** Concentrations of cyclic AMP (cAMP) in H322 cells treated with IL-13 for 8 h $ compared to VEH, *p* < 0.0001. For panels A & B, H322 cells treated with vehicle (*n* = 6) or CREB inhibitor (*n* = 6); for panels C & D, H322 cells treated with vehicle (*n* = 7) or recombinant CREB (n = 8); for panel E, H322 cells treated with vehicle (*n* = 6) or IL-13 (*n* = 8). Abbreviations: IL-13, Interleukin 13; VEH, vehicle; *CREB*, cAMP responsive element binding protein 1.

Due to the cellular complexity of the murine lung, we studied a human cell line (NCI-H322) with characteristics of club cells (Lau et al., 1987; Schuller et al., 1991) to gain more mechanistic insight into the linkage of IL-13 with Creb1 signaling. We first examined the major signaling components of IL-13 and determined their regulation by CREB. We treated NCI-H322 cells with vehicle or the CREB inhibitor 666–15 for four consecutive days to mimic the *in vivo* studies. We measured abundance of mRNA for IL-13 receptors *IL13Rα1, IL13Rα2, and IL4Rα* in NCI-H322 cells. NCI-H322 cells treated with the pharmacologic CREB inhibitor 666–15 showed significantly decreased *IL13Rα1* mRNA expression (*p* = 0.034, Figure 4A) relative to vehicle. CREB inhibition had no effect on *IL4Rα* mRNA expression (Figure 4B). Levels of *IL13Rα2* mRNA were below the threshold of detection in cells treated with vehicle or CREB inhibitor (data not shown).

We also measured *IL13Rα1, IL13Rα2, and IL4Rα* mRNA in NCI-H322 cells treated with recombinant CREB. Recombinant CREB treatment did not modulate *IL13Rα1* mRNA abundance (Figure 4C). However, recombinant CREB treatment reduced expression of *IL4Rα* mRNA by −20% (*p* = 0.0389, Figure 4D). Like the CREB inhibitor studies, levels of *IL13Rα2* mRNA were below the threshold of detection in cells treated with vehicle or recombinant CREB (data not shown).

Lastly, we also measured cAMP levels in H322 cells post IL-13 stimulation. We looked at three different concentrations and two different times points (as described in methods). We found no evidence for increased cAMP levels for any of the concentrations at the 30-min time point (data not shown). Examination of the 8-h time point revealed a significant increase in cAMP concentrations in response to 25 ng/mL IL-13 treatment (*p* < 0.0001, Figure 4E). No changes in cAMP were observed at the 8-h time at the lower concentrations of 6.25 ng/mL and 12.5 ng/mL IL-13 treatment (data not shown). These findings were consistent with our prior work showing a similar increase in cAMP concentrations in response to IL-1B at a delayed time point (Sponchiado et al., 2023) and demonstrated a clear link between IL-13 and CREB signaling.

## Discussion

In this study, we hypothesized that loss of club cell Creb1 would mitigate the pro-mucin effects of IL-13 in mice. We first examined airway mechanics since IL-13 increases airway resistance in part through excess mucin production and obstruction. We found that IL-13 treatment in mice with loss of club cell Creb1 decreased basal total airway resistance and basal Newtonian resistance in a sex-independent manner. Conversely, loss of club cell Creb1 augmented IL-13-mediated increases in total airway elastance in response to methacholine in both male and female airways. Though IL-13 also increased tissue elastance and tissue damping in response to methacholine, those properties were not affected by loss of club cell Creb1*.* The selective effect for loss of club cell Creb1 on airway resistance and airway elastance and not on tissue elastance and tissue damping is consistent with club cells and their function in the airway (Dean and Snelgrove, 2018).

To identify possible explanations for the effect that loss of club cell Creb1 had on airway resistance, we assayed mucin production and secretion in the entire lung. To our surprise we found that loss of club cell Creb1 increased transcription abundance of *Muc5ac* and *Muc5b* mRNA in response to IL-13 in male lungs but not in female lungs. Testosterone decreases type 2 immune responses in an experimental model of allergic asthma in male mice (Fuseini et al., 2018). Thus, one interpretation of our sex-dependent findings is that the beneficial effects of testosterone on type 2 immune responses requires club cell Creb1. Consistent with this, testosterone has been shown to activate Creb1 signaling in other cells (Fix et al., 2004).

Despite sex-independent increases in *Muc5ac* mRNA expression, only male mice showed a trend for increased Muc5ac protein concentrations in the lavage fluid. Further, even though loss of club Creb1 augmented the effect of IL-13 on *Muc5ac* mRNA expression in male lungs, we did not see a corresponding increase in Muc5ac protein concentrations in the bronchoalveolar lavage fluid. Both sexes showed expected IL-13-dependent increases in *Muc5b* protein levels. Even though we observed that loss of club Creb1 augmented IL-13-mediated increases in *Muc5b* mRNA levels in male mice airways, we did not observe a corresponding increase in Muc5b bronchoalveolar lavage fluid concentrations.

The discordance among mRNA expression and protein levels suggested that both IL-13 and loss of club cell Creb1 might have induced deficits in mucin secretion mechanisms. Thus, we measured three molecules important for mucus secretion. IL-13 increased expression of *P2ry2* mRNA in both male and female airways. However, in female airways, loss of club cell Creb1 negated the effect of IL-13 on *P2ry2* mRNA. Finding IL-13-mediated regulation of *P2ry2* receptors has important implications for future preclinical studies since these receptors promote mucin secretion in response to ATP (Hao and Ko, 2014) and are considered pro-inflammatory in allergic asthma models. Though our work and others have demonstrated that IL-1B increases *P2ry2* mRNA (Hou et al., 2000; Sponchiado et al., 2023), upregulation of *P2ry2* in response to IL-13 has not been previously reported. The mechanism responsible for IL-13-mediated upregulation of *P2ry2* mRNA in the lung is unclear, though our data suggest that at least in female airways, part of the effect of IL-13 on *P2ry2* mRNA is club cell Creb1-dependent. This is consistent with prior work indicating that P2ry2 is a target of Creb1 (Rouillard et al., 2016).

We also found that loss of club cell Creb1 decreased whole lung *M3R* mRNA expression in female airways independent of treatment. A similar finding was observed in male airways, with loss of club cell Creb1 decreasing *M3R* mRNA expression. Thus, consistent with our prior work, this finding suggests that of club cell Creb1 signaling may dampen cholinergic-mediated mucin secretion (Sponchiado et al., 2023). Since M3R is also important for airway smooth muscle contraction, it is possible that smooth muscle tone may also have been impacted by loss of club cell Creb1. However, our flexiVent data did not support this possibility.

Lastly, we found that loss of club cell Creb1 tended to increase whole lung mRNA of *Rab3D* in male lungs, with no effect on female lungs. There is at least one paper describing sex-dependent regulation of Rab3D in rodent liver cells (Rajagopal et al., 2020). Cross talk among estrogen signaling and Creb1 signaling has been reported (Berto et al., 2018). More specifically, overexpression of Creb1 promotes induction of estrogen receptor α target genes in a breast carcinoma cell line. If this complex interaction exists in the airway, then some of the sex-dependent differences we observe in our data may be explained in part through Creb1 and estrogen receptor cross talk.

Our whole lung mRNA analysis focused on mucins and mucin secretion machinery lead us to hypothesize that goblet cell density post methacholine stimulation would be impacted by both treatment and genotype. We found that IL-13 treatment increased detection of goblet cells with neutral mucins post methacholine stimulation independent of genotype. Since loss of club cell Creb1 decreased *M3R* mRNA abundance and altered *P2ry2* mRNA abundance, our anticipation was that identification of functional impacts on mucin secretion due to loss of club cell Creb1 may be determined by measuring goblet cell density post methacholine stimulation. Because examination of goblet cell density did not support a functional secretion defect, we also examined surface expression of Muc5b in the lungs. The rationale was that Alcian Blue/PAS staining of mucins in goblet cells does not distinguish among the different types of mucins (secreted and/or tethered) and therefore selective study of a constitutively expressed secreted mucin through antibody labeling might facilitate detection of a secretion defect. In this case, we saw a weak trend for loss of club cell Creb1 to increase surface Muc5b protein in female airways in response to IL-13 treatment. Thus, despite club cell Creb1-mediated regulation of mucin mRNA and mucin secretion machinery, we uncovered no strong evidence that mucin secretion was impacted through the various assays we performed.

We did not see an effect for IL-13 treatment or loss of club cell Creb1 on the density of goblet cells expressing acidic mucins post methacholine stimulation. Thus, one interpretation of this findings is that secretion of acidic mucins in not impacted by IL-13 or loss of club cell Creb1. While others have reported that IL-13 increases production of both acidic and neutral mucins (Zhu et al., 1999), we reported a similar selectivity for goblet cells with neutral mucins post methacholine stimulation in response to IL-1B treatment (Sponchiado et al., 2023). Another speculation is that ratio of acidic mucins to neutral mucins is higher in the airways of mice following repeated IL-13 administration. Though people with cystic fibrosis have higher amounts of acidic mucins (Xia et al., 2005), and a greater concentrations of neutral mucins has been reported in people with chronic obstructive pulmonary disorder (Caramori et al., 2004), there is a lack of evidence to suggest that people with asthma have higher concentrations of acid mucins in their airways.

Finally, we performed studies in a human “club cell-like” cell line (NCI-H322) to investigate the link between IL-13 and CREB1. We established that NCI-H322 cells expressed key receptors for IL-13 signaling, including IL13Rα1 and IL4Rα*.* Signal transduction pathway through the IL-4Rα and IL-13Rα1 chains is characteristic of a type II IL-13 signaling*.* This type II receptor complex is found on many non-hematopoietic cells, such as airway epithelial cells, fibroblasts and smooth muscle cells (Akaiwa et al., 2001), and predominantly activates STAT6 (Marone et al., 2019). STAT6 is a well-established target of the CREB signaling complex (Zhang et al., 2011). Surprisingly, we also found that CREB1 differentially regulated these receptors, with inhibition of CREB1 decreasing *IL13Rα1* mRNA expression and overexpression of CREB1 inhibiting *IL4Rα* mRNA expression. Because no studies have reported cAMP response element binding sites in the promoter regions of either *IL13Rα1* or *IL4Rα*, one speculation is that CREB1-mediated regulation of these receptors is indirect. We also established that application of IL-13 to NCI-H322 cells increased cAMP concentrations in a delayed manner. The delayed manner suggested an intermediary signaling pathway. Consistent with this, we and others have reported similar findings for IL-1B, in which increases in cAMP were delayed and attributed to prostaglandins (Gray et al., 2004). Thus, it is possible a similar mechanism is in place for IL-13 mediated increases in cAMP concentrations.

Our study has some limitations. We observed several sex differences in our murine studies but did not uncover the mechanisms responsible for sex differences. Thus, future studies focused on mechanisms to explain sex-differences are warranted. Our studies in mice were also focused on whole lung physiology and whole lung biology. This global approach is valuable because it provides the broadest view of functional and cellular deficits or corrections in our experimental model system. However, our interpretations are somewhat limited because we do not know the exact cell type or population that may be driving some of the changes we observed. Thus, more refined approaches such as single cell RNA-sequencing or lineage tracing studies may provide more granular and mechanistic insight. Lastly, NCI-H322 cells are from a male donor. We are unaware of a similar cell line from a female donor. Thus, it is possible that the relationship we revealed in the NCI-H322 cell line linking IL-13 signaling to CREB1 may be different in a human “club cell-like” line derived from females.

In summary, our study identified club cell Creb1 as a regulator of airway mechanics and mucin secretion mechanisms in response to IL-13. In some cases, this regulation was sex-independent, and in other cases, sex-dependent. Thus, these studies enhance our understanding of the cellular mechanisms contributing to IL-13-mediated airway pathophysiology and shed new light onto sex specific differences.

## Data Availability

The original contributions presented in the study are included in the article/Supplementary Material, further inquiries can be directed to the corresponding author.
