# Supplementary material for: Sex-dependent regulation of mucin gene transcription and airway secretion and mechanics following intra-airway IL-13 in mice with conditional loss of club cell Creb1
Source: Front Physiol. 2024 Apr 22;15:1392443. doi: 10.3389/fphys.2024.1392443 (PMC11070562; doi:10.3389/fphys.2024.1392443)
Supplement: Supplementary file 1 [file Table1.docx]

**Supplemental Table S1.** Primer pairs used for quantitative real-time PCR analyses.

| **Gene Symbol** | **Gene** | **GenBank accession** | **Sequence of Forward (5’-3’) and Reverse (3’-5’) primers** | **Amplicon (bp)** |
| --- | --- | --- | --- | --- |
| *Muc5b* | mucin 5, subtype B, tracheobronchial | NM_028801.2 | 5’ ACATCCTGACCAAGAAATGTGC  3’ GACAAGGGCATCTGCGTAAAG | 190 |
| *Muc5ac* | mucin 5, subtypes A and C, tracheobronchial/gastric | NM_010844.3 | 5’ GTGGTGGAAACTGACATTGG  3’ CATCAAAGTTCCCACACAGG | 115 |
| *Cre* | Cre recombinase | - | 5’ TGCCTGCATTACCGGTCG  3’GCATAACCAGTGAAACAGCATTGCTG | 321 |
| *Rab3D* | member RAS oncogene family (Rab3d) | [NM_031874.5](https://www.ncbi.nlm.nih.gov/nucleotide/NM_031874.5?report=genbank&log$=nuclalign&blast_rank=1&RID=GYXV00VS013) | 5’ CGAGATCCACGTGTCGGAAG  3’ CACTAGCGGATGCCATCTCA | 312 |
| *P2ry2* | Purinergic receptor P2Y, G-protein coupled receptor 2 | NM_008773.4 | 5’AGCCCATTACGTGACTGTCCCGAG  3’ CCGGAGGACTCCGAGATCA | 128 |
| *M3R* | cholinergic receptor, muscarinic 3 | NM_033269.4 | 5’CACGAGCGAACCTGAGGAC  3’ATGCCATTGCTGGTCATATCTGG | 162 |
| *Actb* | actin, beta | NM_007393.5 | 5’ CTGTGGCATCCATGAAACTACA  3’ GTAATCTCCTTCTGCATCCTGTCA | 141 |
| *RPL13A* | ribosomal protein L13a | NM_012423.4 | 5’ GGCCCCTACCACTTCCG  3’ ACTGCCTGGTACTTCCA | 251 |
| *IL13Rα1* | interleukin 13 receptor subunit alpha 1 | NM_001560.3 | 5’ AGGAATACCAGTCCCGACAC  3’ TGGAATCCTTCACTTTGGTC | 138 |
| *IL13Rα2* | interleukin 13 receptor subunit alpha 2 | NM_000640.3 | 5’ TCTTGGAAACCTGGCATAGG  3’ TGCCTCCAAATAGGGAAATC | 146 |
| *IL4Rα* | interleukin 4 receptor | NM_000418.4 | 5’ AACGACCCGGCAGATTTCAG  3’ AGGAGTTGTGCCACTTGGTG | 174 |
