# Supplementary material for: Sex-dependent regulation of mucin gene transcription and airway secretion and mechanics following intra-airway IL-13 in mice with conditional loss of club cell Creb1
Source: Front Physiol. 2024 Apr 22;15:1392443. doi: 10.3389/fphys.2024.1392443 (PMC11070562; doi:10.3389/fphys.2024.1392443)
Supplement: Supplementary file 2 [file Image1.pdf]

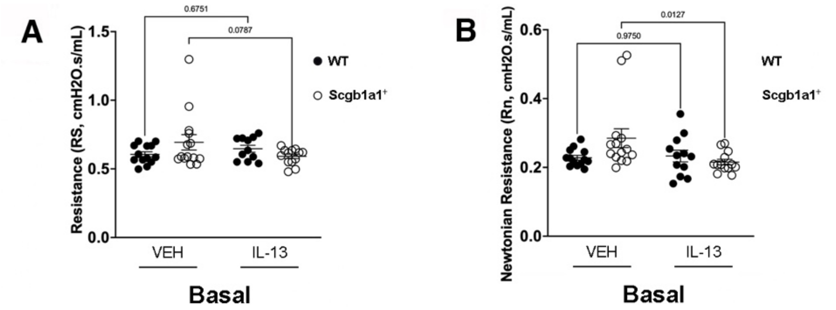

Supplemental Figure S1. (A) Demonstration of treatment x genotype interaction in basal airway resistance. This interaction meant that when collapsing data across sexes, loss of club cell *Creb1* influenced the impact of IL-13 on basal airway resistance. (B) Demonstration of treatment x genotype interaction on basal Newtonian resistance. This meant that genotype influenced the effect of IL-13 when data were collapsed across sexes. $Creb1^{fl/fl}Scgb1a1^{wt}$ mice treated with vehicle (n = 13) or IL-13 (n = 12); $Creb1^{fl/fl}Scgb1a1^{+}$ mice treated with vehicle (n = 14) or IL-13 (n = 13). Abbreviations: WT, wild type; $Scgb1a1^{+}$, club cell promoter driving CRE recombinase; IL-13, Interleukin 13; VEH, vehicle
